# ﻿Phylogeny and taxonomy of two new species in Dictyosporiaceae (Pleosporales, Dothideomycetes) from Guizhou, China

**DOI:** 10.3897/mycokeys.106.125693

**Published:** 2024-06-27

**Authors:** Yao Feng, Zuo-Yi Liu, Xiao-Fang Chen, Mi-Lian Yang, Zhi-Yuan Zhang, Ya-Ya Chen

**Affiliations:** 1 School of Chinese Ethnic Medicine, Guizhou Minzu University, Guiyang, Guizhou 550025, China; 2 Guizhou Key Laboratory of Agricultural Biotechnology, Guizhou Academy of Agricultural Sciences, Guiyang, Guizhou 550025, China; 3 Bijie Medical College, Bijie, Guizhou 551700, China; 4 College of Eco-Environmental Engineering, Guizhou Minzu University, Guiyang, Guizhou 550025, China; 5 Institute of Crop Germplasm Resources, Guizhou Academy of Agricultural Sciences, Guiyang, Guizhou 550025, China; 6 Ministry of Agriculture and Rural Affairs, Key Laboratory of Crop Cenetic Resources and Germplasm Innovation in Karst Region, Guiyang, Guizhou 550009, China

**Keywords:** 2 new species, *Gregarithecium*, multi-locus, new taxa, *Pseudocoleophoma*, taxonomy

## Abstract

Two novel species within the family Dictyosporiaceae are described and illustrated from terrestrial habitats on dead culms of bamboo and an unidentified plant, respectively. Through morphological comparisons and the multi-locus phylogenetic analyses of combined LSU, ITS, SSU, and *tef1-α* sequence dataset, two species, *Gregaritheciumbambusicola*, *Pseudocoleophomaparaphysoidea* are identified. Phylogenetically, both species clustered into a monophyletic clade with strong bootstrap support. *Gregaritheciumbambusicola***sp. nov.** can be distinguished from other species within the genus based on its almost straight ascospores. *Pseudocoleophomaparaphysoidea***sp. nov.** differs from other species in its conidiogenous cells intermixed with paraphyses, longer conidiogenous cells and larger conidia. The identification of this lineage contributes to our understanding of the classification of Dictyosporiaceae.

## ﻿Introduction

Boonmee et al. (2016) established the family Dictyosporiaceae, with the type genus *Dictyosporium*, based on morphology and multi-locus phylogenetic analysis. Members of Dictyosporiaceae are mostly saprobic, globally distributed and commonly found in terrestrial and aquatic habitats (Boonmee et al. 2016, 2021). The main diagnostic criteria of Dictyosporiaceae are immersed to erumpent or superficial, subglobose to globose, dark brown to black ascomata, bitunicate asci with septate, hyaline, sheathed ascospores; the asexual morphs are cheirosporous hyphomycetes (Boonmee et al. 2016; Tennakoon et al. 2019; Hongsanan et al. 2020). Currently, Dictyosporiaceae comprises 18 genera (Hongsanan et al. 2020; Tian et al. 2022; Wijayawardene et al. 2022).

Tanaka et al. (2015) erected the genus *Gregarithecium* and *Pseudocoleophoma* within Dictyosporiaceae, with *Gregaritheciumcurvisporum* and *Pseudocoleophomacalamagrostidis* as the type species, respectively. *Gregarithecium* is characterized by immersed to erumpent, grouped ascomata with fissitunicate, cylindrical, short-stalked asci, broadly fusiform, hyaline ascospore with a median septum, surrounded by an entire sheath (Tanaka et al. 2015; Tennakoon et al. 2019; Lu et al. 2022). *Pseudocoleophoma* is characterized by ostiolar ascomata; brown and polygonal to rectangular cells of peridium; cylindrical to clavate and fissitunicate asci with numerous pseudoparaphyses; fusiform, and septate ascospores, with an apparent sheath (Tanaka et al. 2015; Lu et al. 2022). The asexual morph of *Pseudocoleophoma* is pycnidial, which is characterized by subglobose conidiomata, doliiform and phialidic conidiogenous cells, and cylindrical or oblong, hyaline, aseptate, smooth-walled conidia (Jiang et al. 2021). Currently, only one species is accepted in the genus *Gregarithecium*, while *Pseudocoleophoma* has 13 records listed in Index Fungorum.

In this study, we introduce two new taxa (*Gregaritheciumbambusicola* and *Pseudocoleophomaparaphysoidea*) belonging to Dictyosporiaceae, collected from landscape plants in Guizhou Province, China. Morphological observations and phylogenetic analyses were conducted to clarify the classification of these species and their evolutionary relationships with closely related species. Detailed descriptions of the morphological features of these species along with their molecular characterization are provided.

## ﻿Materials and methods

### ﻿Sample collection, Fungal isolation and morphological studies

Fresh fungal specimens were collected in Guizhou Province, China. The specimens were examined by using a stereomicroscope (Motic SMZ 168). Freehand sections of ascomata and other fungal structures were photographed using a Nikon ECLIPSE Ni compound microscope fitted with a Nikon DS-Ri2 digital camera. Measurements for all structural components were made with Tarosoft Image FrameWork software (IFW 0.97 version) (Liu et al. 2010). Single spore isolations were carried out following the approaches in Chomnunti et al. (2014). Type specimens were deposited in the herbarium of Guizhou Academy of Agriculture sciences (GZAAS), Guiyang, China. All living cultures were stored in a metabolically inactive state (i.e., kept in sterile 30% glycerol in a –80 °C freezer), which were deposited in Guizhou Culture Collection (GZCC), Guiyang, China. Facesoffungi (http://www.facesoffungi.org/) numbers were obtained as in Jayasiri et al. (2015). The new species are registered in Index Fungorum (2024, http://www.indexfungorum.org/).

### ﻿DNA extraction, PCR amplification and sequencing

Fungal mycelia were scraped with a surgical knife from the pure culture which was growing on potato dextrose agar (PDA) for one week at 25 °C in dark. The total genomic DNA was conducted by using Ezup Column Fungi Genomic DNA Purification Kit (Sangon Biotech, China) from fresh fungal mycelia. Four gene regions, internal transcribed spacer (ITS), large subunit rDNA (LSU), small subunit rDNA (SSU) and the translation elongation factor 1-alpha (*tef1-α*) were amplified and sequenced using primers listed in Table 1. Polymerase chain reaction (PCR) was carried out in a 25 μL reaction volume, which contained 12.5 μL 2 × PCR Master Mix (Sangon Biotech, China), 8.5 μL ddH_2_O, 1 μL of each primer and 2 μL DNA template. The amplification conditions for all four loci consisted of initial denaturation at 95 °C for 5 min; followed by 35 cycles of 1 min at 94 °C, 1 min at 52 °C, and 1.5 min at 72 °C, and a final extension period of 10 min at 72 °C. PCR products were analyzed using 1.2% agarose electrophoresis gel stained with ethidium bromide and sequenced by Sangon Biotech (Shanghai) Co., Ltd, China. New generated nucleotide sequences were submitted in GenBank (Table 2).

**Table 1.** Sequences of primers used in this study.

Molecular marker	Primer name	Primer sequence (5´–3´)	Reference
SSU	NS1	GTAGTCATATGCTTGTCTC	White et al. 1990
	NS4	CTTCCGTCAATTCCTTTAAG	White et al. 1990
ITS	ITS1 (*Gregarithecium*)	TCCGTAGGTGAACCTGCG	White et al. 1990
	ITS4	TCCTCCGCTTATTGATATGC	White et al. 1990
	ITS5 (*Pseudocoleophoma*)	GGAAGTAAAAGTCGTAACAAGG	White et al. 1990
LSU	LR5	ATCCTGAGGGAAACTTC	Vilgalys and Hester 1990
	LR0R	ACCCGCTGAACTTAAGC	Vilgalys and Hester 1990
*tef1-α*	983F	GCYCCYGGHCAYCGTGAYTTYAT	Rehner and Buckley 2005
	2218R	ATGACACCRACRGCRACRGTYTG	Rehner and Buckley 2005

**Table 2.** GenBank accession numbers of the sequences used in this study.

Taxa	Voucher/Culture	GenBank accession numbers			
		LSU	ITS	SSU	*tef1-α*
*Aquadictyosporaclematidis*	MFLUCC 17-2080	MT214545	MT310592	MT226664	MT394727
*Aquadictyosporalignicola*	MFLUCC 17-1318 **T**	MF948629	MF948621	–	MF953164
*Aquaticheirosporalignicola*	HKUCC 10304	AY736378	AY864770	AY736377	–
*Cheirosporiumtriseriale*	HMAS 180703 **T**	EU413954	EU413953	–	–
*Dendryphiellafasciculata*	MFLUCC 17-1074 **T**	MF399214	MF399213	–	–
*Dendryphiellaparavinosa*	CBS 141286 **T**	KX228309	KX228257	–	–
*Dictyocheirosporabannica*	KH 332	AB807513	LC014543	–	AB808489
*Dictyocheirosporapseudomusae*	yone 234	AB807520	LC014550	AB797230	AB808496
*Dictyocheirosporarotunda*	MFLUCC 14-0293 **T**	KU179100	KU179099	KU179101	–
*Dictyosporiumappendiculatum*	KUMCC 17-0311	MH376715	MH388343	–	–
*Dictyosporiumdigitatum*	KUMCC 17-0269	MH376716	MH388344	MH388311	MH388378
*Dictyosporiumguttulatum*	KUMCC 17-0288	MH376717	MH388345	MH388312	MH388379
*Dictyosporiumhongkongensis*	KUMCC 17-0268	MH376718	MH388346	MH388313	MH388380
*Digitodesmiumchiangmaiense*	KUN-HKAS 102163	MK571766	–	MK571775	–
*Digitodesmiumpolybrachiatum*	CoAD 3175	MW879317	MW879319	MW879326	–
*Digitodesmiumpolybrachiatum*	COAD 3174	MW879316	MW879318	MW879325	–
***Gregaritheciumbambusicola***	**GZCC 21-0713 T**	**PP639379**	**PP639375**	**PP661224**	**PP624323**
***Gregaritheciumbambusicola***	**GZCC 21-1120**	**PP639380**	**PP639376**	**PP661225**	**PP624324**
*Gregaritheciumcurvisporum*	KT 922 **T**	AB807547	AB809644	AB797257	AB808523
*Immotthiabambusae*	KUNHKAS 112012 **T**	MW489450	MW489455	MW489461	MW504646
*Jalapriyapulchra*	MFLUCC 15-0348 **T**	KU179109	KU179108	KU179110	–
*Jalapriyapulchra*	MFLUCC 17-1683	MF948636	MF948628	–	MF953171
*Murilentitheciumclematidis*	MFLUCC 14-0561	KM408758	KM408756	–	KM454444
*Murilentitheciumclematidis*	MFLUCC 14-0562 **T**	KM408759	KM408757	NG_061185	KM454445
*Neodendryphiellamali*	CBS 139.95 **T**	LT906657	LT906655	–	–
*Neodigitodesmiumcheirosporum*	UESTCC 22.0020	ON595713	ON595714	ON595712	ON595700
*Pseudocoleophomabauhiniae*	MFLUCC 17-2580	MK347952	MK347735	MK347843	MK360075
*Pseudocoleophomabauhiniae*	MFLUCC 17-2586 **T**	MK347953	MK347736	MK347844	MK360076
*Pseudocoleophomacalamagrostidis*	KT 3284 **T**	LC014609	LC014592	LC014604	LC014614
*Pseudocoleophomaclematidis*	MFLUCC 17-2177 **T**	NG_073844	MT310596	MT226667	MT394730
*Pseudocoleophomaflavescens*	CBS 178.93	GU238075	–	GU238216	–
*Pseudocoleophomaguizhouensis*	MFLU 18-2262	OP099522	OR225073	OR134444	OR140434
*Pseudocoleophomaheteropanacicola*	ZHKUCC 23-0880 **T**	OR365486	OR365486	–	OR700204
***Pseudocoleophomaparaphysoidea***	**GZCC 21-0711 T**	**PP639377**	**PP639373**	**PP661222**	**PP624321**
***Pseudocoleophomaparaphysoidea***	**GZCC 21-0712**	**PP639378**	**PP639374**	**PP661223**	**PP624322**
*Pseudocoleophomapolygonicola*	KT 731 **T**	AB807546	AB809634	AB797256	–
*Pseudocoleophomapuerensis*	ZHKUCC 22-0204 **T**	OP297769	OP297799	OP297783	OP321568
*Pseudocoleophomapuerensis*	ZHKUCC 22-0205	OP297770	OP297800	OP297784	OP321569
*Pseudocoleophomarhapidis*	ZHKUCC 21-0124 **T**	ON244661	ON244664	ON244667	–
*Pseudocoleophomarusci*	MFLU 16-0292	MT183514	MT185549	MT214983	–
*Pseudocoleophomarusci*	MFLUCC 16-1444 **T**	NG_073840	NR_170045	NG_070346	–
*Pseudocoleophomatyphicola*	MFLUCC 16-0123 **T**	KX576656	KX576655	–	–
*Pseudocoleophomayunnanensis*	ZHKUCC 22-0200 **T**	OP297765	OP297795	OP297779	OP321564
*Pseudocoleophomayunnanensis*	ZHKUCC 22-0201	OP297766	OP297796	OP297780	OP321565
*Pseudocoleophomazingiberacearum*	NCYUCC 19-0054 **T**	MN616755	MN615941	–	MN629283
*Pseudoconiothyriumbroussonetiae*	CPC 33570	NG_066331	NR_163377	–	–
*Pseudodictyosporiumthailandica*	MFLUCC 16-0029 **T**	KX259522	KX259520	–	KX259526
*Pseudodictyosporiumwauense*	NBRC 30078	DQ018105	DQ018098	–	–
*Pseudodictyosporiumwauense*	DLUCC 0801	MF948630	MF948622	–	MF953165
*Verrucoccumcoppinsii*	SPO 2343	MT918765	MT918780	MT918773	–
*Verrucoccumhymeniicola*	CBS 845.96	AB807567	LC014586	AB797277	AB808543
*Vikalpaaustraliensis*	HKUCC 8797 **T**	–	DQ018092	–	–

Notes: “**T**” stands for Ex-type strains. Sequences highlighted in bold were generated in this study.

### ﻿Phylogenetic analyses

Phylogenetic analyses of Dictyosporiaceae were performed based on ITS, LSU, SSU, and *tef1-α* sequence data. The representative taxa of Dictyosporiaceae (Table 2) were referred to BLAST (https://blast.ncbi.nlm.nih.gov/Blast.cgi) result and relevant publications (Lu et al. 2022; Tian et al. 2022). Sequences were aligned using MAFFT v. 7 (Katoh and Standley 2013). Manual adjustments were performed when it is necessary using BioEdit v. 7.0 (Hall 1999). Phylogenetic analyses of maximum likelihood (ML) and Bayesian inference (BI) were conducted based on combined datasets.

ML analysis was performed with raxmlGUI v. 1.3 (Silvestro and Michalak 2012) and the topology was evaluated using 1,000 ultrafast bootstrap replicates. The phylogenetic analyses were performed for Bayesian inference in MrBayes 3.2.6. The model of evolution was estimated by ModelTest 2. The Markov chain Monte Carlo (MCMC) sampling in MrBayes 3.2.6 was used to determine the posterior probabilities (PP). Every 100^th^ generation was sampled as a tree with 1,000,000 generations running for six MCMC chains (Huelsenbeck and Ronquist 2001; Zhaxybayeva and Gogarten 2002; Nylander 2004). Phylogenetic trees were viewed with FigTree v1.4.2 (Rambaut 2012) and edited using Adobe Illustrator 2021 (2.6.0.44) and Adobe Photoshop CS6 software (Adobe Systems, USA).

## ﻿Results

### ﻿Phylogenetic analyses

**Figure 1.**
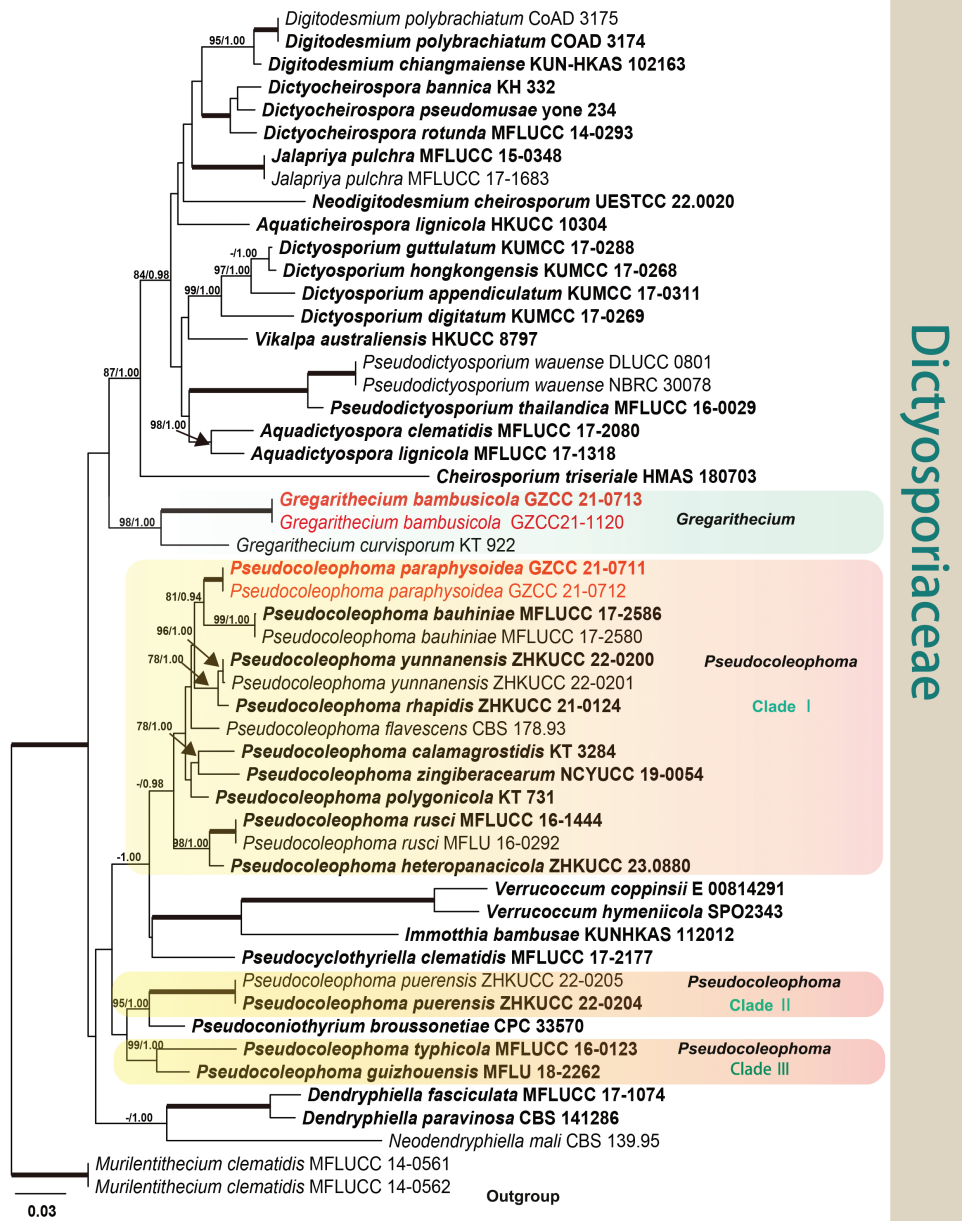
Phylogram based on the maximum likelihood (ML) analysis using the LSU, ITS, SSU, and *tef1-α* sequences of Dictyosporiaceae. Bootstrap support values for ML equal to or greater than 75% and the Bayesian posterior probabilities equal to or higher than 0.95 PP are indicated above the nodes as ML/PP. Ex-type strains are in black bold and the new taxa are highlighted in bold and red.

To determine the phylogenetic placement of the new taxa within the family Dictyosporiaceae, a dataset consisting of combined LSU, ITS, SSU, and *tef1-α* sequences was analyzed, including a total of 52 taxa. *Murilentitheciumclematidis* (MFLUCC 14-0561 and MFLUCC 14-0562) was used as the outgroup taxa for the analysis. The concatenated alignment comprises 3,376 characters (LSU: 1–803; ITS: 804–1,340; SSU: 1,341–2,338; *tef1-α*: 2,339–3,376) including gaps. Maximum likelihood and Bayesian analyses were performed, respectively, and presented consistent topologies. Bayesian posterior probabilities were calculated with a final average standard deviation of split frequencies of less than 0.01. The best scoring RAxML tree (Fig. 1) was built with a final likelihood value of -18784.512555. Estimated base frequencies were as follows: A = 0.236875, C = 0.246782, G = 0.270789, T = 0.245554; substitution rates AC = 1.688035, AG = 3.527408, AT = 2.540885, CG = 1.033994, CT = 9.137618, GT = 1.000000. The gamma distribution shape parameter alpha is equal to 0.178657 and the Tree-Length equal to 2.160218.

### ﻿Taxonomy

#### ﻿ Gregarithecium bambusicola

Taxon classificationFungiPleosporalesDictyosporiaceae

Y. Feng & Z. Y. Liu sp. nov.

EB59D730-9C84-5B2C-8DE4-64CD5FE81151

Index Fungorum: IF901991

Facesoffungi Number: FoF15871

Fig. 2

##### Etymology.

The epithet refers to the species inhabiting on bamboo.

##### Holotype.

GZAAS 21-0199.

##### Diagnosis.

***Saprobic*** on dead bamboo culms, the surface of the host has a withered spot with a central protrusion. **Sexual morph: *Ascomata*** 386–658 × 129–237 μm (av. 487 × 169 μm, n= 10), scattered to gregarious, immersed with only ostiolar necks visible on the host surface or erumpent, globose to hemispherical with flattened base in section. ***Peridium*** composed of several layers of hyaline to dark brown cells of ***textura angularis***. ***Hamathecium*** comprising dense, hyaline, branched and anastomosed, septate pseudoparaphyses. ***Asci*** 75–104 µm × 17–26 µm (av. 91 × 20 μm, n = 10), 8- spored, cylindrical, fissitunicate, rounded at the apex with a shallow ocular chamber, small stalk at the base. ***Ascospores*** 25–27 × 5–7 μm (av. 26 × 6 μm, n = 10), biseriate, fusiform, hyaline, mostly straight, septum and constricted, smooth, guttulate, with a distinct gelatinous sheath. **Asexual morph**: undetermined.

**Figure 2.**
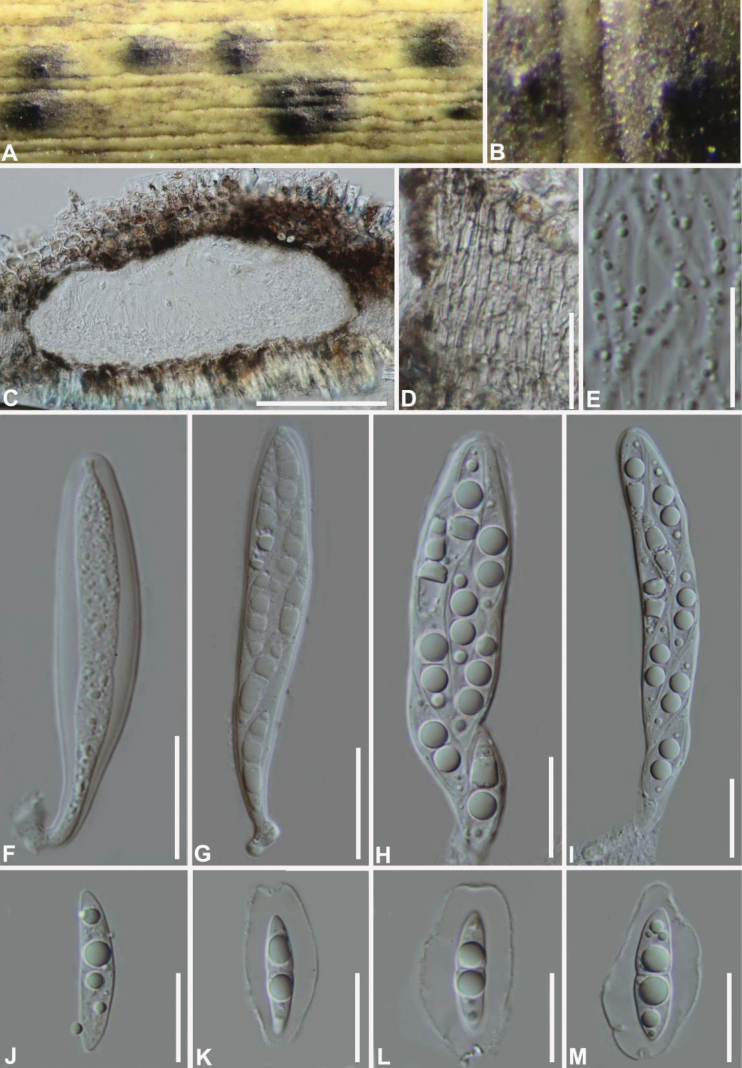
Morphology of *Gregaritheciumbambusicola* (GZAAS 21-0199, holotype) **A, B** appearance of ascomata on host **C** vertical section of ascoma **D** peridium **E** pseudoparaphyses **F–I** asci **J–M** ascospores. Scale bars: 50 μm (**C**); 30 μm (**D**); 20 μm (**F–I**); 10 μm (**E, J−M**).

##### Culture characteristics.

***Ascospores*** germinating on WA within 12 h. Colonies slow growing on PDA at 25 °C, reaching 2 cm diam. in 1 week at 25 °C. Colonies irregular circular, entire edge, white, off-white in reverse.

##### Material examined.

China, Guizhou Province, Xingyi City, on dead culms of bamboo, 2 May 2019, Yao Feng, XY-40 (holotype GZAAS 21-0401, ex-type living culture GZCC 21-1120), *ibid.*, XY-40b (isotype GZAAS21-0401, living culture GZCC21-1120).

##### Notes.

The genus *Gregarithecium* comprises a single species, *G.curvisporum*, which was collected from the culms of *Sasa* sp. (Tanaka et al. 2015). In this study, two new strains clustered in a single clade with high support value (98/1.00), and were closely related to *G.curvisporum* (Fig. 1). *Gregaritheciumbambusicola* resembles the type species in having cylindrical asci and transparent fusiform, guttulate ascospores surrounded by an entire sheath (Tanaka et al. 2015). However, unlike the curved ascospores observed in *G.curvisporum*, *G.bambusicola* has predominantly straight ascospores (Tanaka et al. 2015). Furthermore, the ascospores of *G.curvisporum* have three septa after maturation, which is not seen in *G.bambusicola* (Tanaka et al. 2015). In addition, they can be distinguished by their low sequence similarities. In a comparison of LSU, ITS, SSU, and *tef1-α* nucleotides, *G.bambusicola* (Type strain GZCC 21-0713) has 98%, 87%, 99% and 94% similarity, in LSU (782/800 bp, 2 gaps), ITS (420/484 bp, 5 gaps), SSU (527/534 bp, no gap), and *tef1-α* (780/834 bp, no gap), which is different from *G.curvisporum* (Type strain KT 922).

#### ﻿ Pseudocoleophoma paraphysoidea

Taxon classificationFungiPleosporalesDictyosporiaceae

Y. Feng & Z. Y. Liu sp. nov.

D0DA9237-7753-56F2-B3D2-8C667EC4F10E

Index Fungorum: IF901990

Facesoffungi Number: FoF15872

Fig. 3

##### Etymology.

The epithet refers to the species having paraphyses.

##### Holotype.

GZAAS 21-0197.

##### Diagnosis.

***Saprobic*** on decaying wood in terrestrial habitats, and immersed in host epidermis. At maturity, the fruiting body breaks through host epidermis. **Sexual morph**: undetermined. **Asexual morph: *Conidiomata*** dark brown to black, pycnidial, solitary to gregarious, globose to subglobose, apapillate, ostiolate. ***Conidiomatal wall*** comprising several layers of cells of ***textura angularis***, with inner layers comprising hyaline to dark brown and outer layers composed of dark brown to black cells. ***Conidiogenous cells*** 11–27 × 3–5 μm (av. 21 × 4 μm, n = 20), hyaline, enteroblastic, phialidic, with minute collarette, doliiform, ampulliform, arising from the innermost layer of the pycnidial wall, intermixed with hyaline, filamentous, septate paraphyses. ***Conidia*** 12–15 (−23) × 2–4 μm (av. 14 × 3 μm, n = 30), hyaline, smooth, cylindrical to subcylindrical or fusiform, straight or slightly curved, aseptate, guttulate at both ends.

##### Culture characteristics.

***Conidia*** germinating on WA within 12 h and germ tubes produced from the basal end. After transfer to the PDA, the colonies grew rapidly, reaching 5 cm diam. in 1 week at 25 °C. Part of the mycelia grew on the surface of the medium, compact, violet, with a light-colored rim, and part of the mycelia remained immersed in the medium. The central area of the colony on the back was reddish-brown, the middle white, and the edge light-colored.

**Figure 3.**
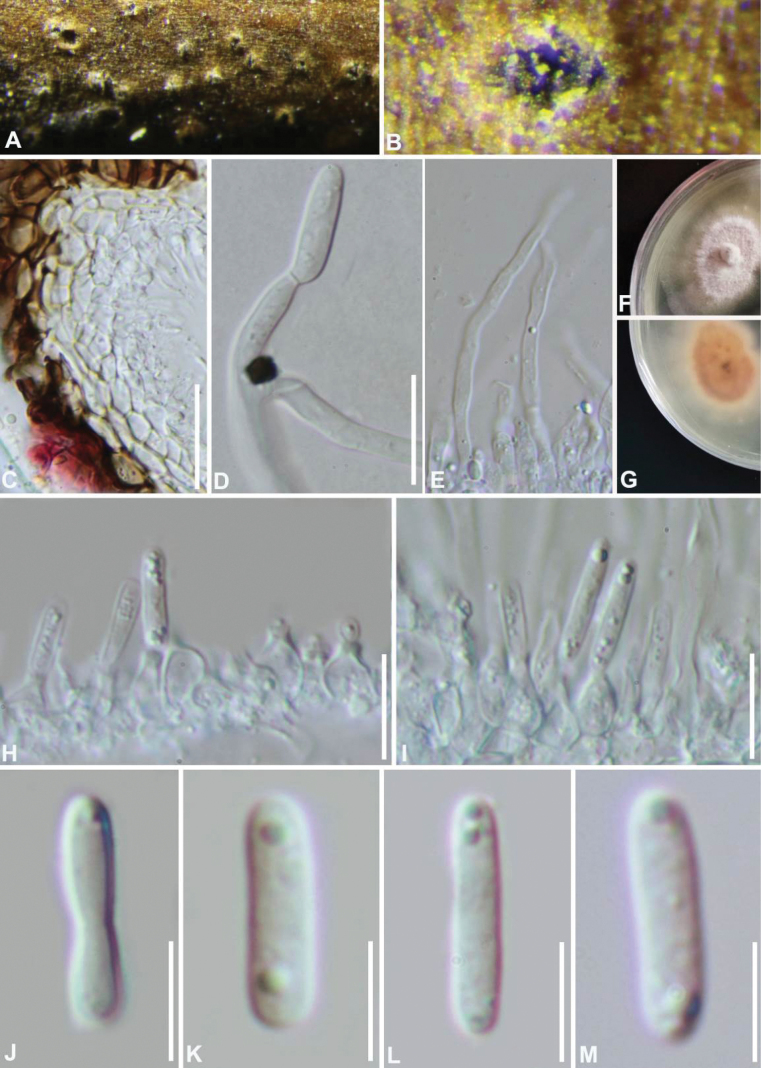
Morphology of *Pseudocoleophomaparaphysoidea* (GZAAS 21-0197, holotype) **A, B** appearance of pycnidia on host **C** peridium **D** germinating conidium **E** paraphyses **F, G** culture **H, I** conidiogenous cells and conidia **J–M** conidia. Scale bars: 20 μm (**C**); 10 μm (**D, H, I**); 5 μm (**J–M**).

##### Material examined.

China, Guizhou Province, Guiyang City, Guizhou Academy of Agricultural Sciences, on dead culms of an unidentified plant, 18 June 2018, Zuo-Peng Liu, NK-1 (holotype GZAAS 21-0197, ex-type living culture GZCC 21-0711). China, Guizhou Province, Xingyi City, on dead culms of an unidentified plant, 7 August 2019, Yao Feng XY19-13 (paratype GZAAS 21-0198, living culture GZCC 21-0712).

##### Notes.

The multi-locus phylogenetic analyses showed that the new isolates GZCC 21-0711 and GZCC 21-0711 (*Pseudocoleophomaparaphysoidea*) formed a single clade and clustered together with high support value 81/0.94 (Fig. 1). *Pseudocoleophomaparaphysoidea* differs from *P.bauhiniae* in its conidiogenous cells intermixed with paraphyses, longer conidiogenous cells (11–27 × 3–5 μm vs. 2.5–5.5 × 2–3 µm) and larger conidia (12–15 × 2–4 μm vs. 7.5–11 × 2–3 µm) (Jayasiri et al. 2019). In addition, they can be distinguished by their low sequence similarities. In a comparison of LSU, ITS, SSU, and *tef1-α* nucleotides, *P.paraphysoidea* (type strain GZCC 21-0711) has 99%, 97%, 99% and 93% similarity, in LSU (789/795 bp, no gap), ITS (490/503 bp, 2 gaps), SSU (1008/1010 bp, one gap), and *tef1-α* (815/878 bp, no gap), which is different from *P.bauhiniae* (Type strain MLFUCC 17-2586).

### ﻿Key to the genus *Pseudocoleophoma*

**Table:** 

1	Asexual and sexual morph produced	**2**
–	Asexual or sexual morph produced	**3**
2	Ascospores 1-septate, with sheath	**4**
–	Ascospores 1–3-septate, without sheath	***P.bauhiniae***
3	Asexual morph produced	**5**
–	Sexual morph produced	**6**
4	Ascomata 160–220 × 140–200 µm, scattered	***P.calamagrostidis***
–	Ascomata 280–350 × 230–310 µm, scattered to 2–4-gregarious	***P.polygonicola***
5	Conidia aseptate	**7**
–	Conidia 1-euseptate	***P.typhicola***
6	Ascospores fusiform	**8**
–	Ascospores narrowly ellipsoid or oblong	***P.puerensis***
7	Conidiomata ostiolate	**9**
–	Conidiomata apapillate, ostiole	**10**
8	Ascospores 1-septate	**11**
–	Ascospores 3-septate	***P.heteropanacicola***
9	Conidia 20–25 × 10–15 µm, oblong to obovoid	***P.rhapidis***
–	Conidia 8–14 × 3–6 µm, cylindrical to subcylindrical or fusiform	***P.rusci***
10	Conidiomata solitary to gregarious	**12**
–	Conidiomata solitary	***P.zingiberacearum***
11	Ascomata gregarious, scattered; Asci clavate	***P.guizhouensis***
–	Ascomata solitary or scattered; Asci clavate to cylindrical, fissitunicate	***P.yunnanensis***
12	Conidiogenous cells globose to doliiform; conidia ellipsoidal ***P.flavescens***
–	Conidiogenous cells doliiform, ampulliform; conidia cylindrical to subcylindrical or fusiform	***P.paraphysoidea***

## ﻿Discussion

In this study, *Gregaritheciumbambusicola* and *Pseudocoleophomaparaphysoidea* are described as two new species in Dictyosporiaceae based on phylogenetic analysis and morphological features. The phylogenetic analysis revealed their distinct genetic relationships and their placement within the family Dictyosporiaceae. Dictyosporiaceae has a diverse species distribution in dead leaves of *Alauraceous* tree, leaves of *Citrussinensis*, submerged wood, soil and other hosts. The discovery of the new species is of great significance to the species diversity, classification and geographical distribution of Dictyosporiaceae.

The genus *Pseudocoleophoma* has 13 species in Index Fungorum. Among these species, four species were described based on the sexual morph (*viz. P.guizhouensis*, *P.heteropanacicola*, *P.puerensis*, and *P.yunnanensis*), five species were based on asexual morph (*viz. P.flavescens*, *P.typhicola*, *P.rhapidis*, *P.rusci*, and *P.zingiberacearum*), only three species have been reported for both the holomorphs (*viz. P.bauhiniae*, *P.calamagrostidis*, and *P.polygonicola*) (Tanaka et al. 2015; Jayasiri et al. 2019; Jiang et al. 2021). Jiang et al. (2021) synonymized *Pseudocoleophomaclematidis* as *Pseudocyclothyriellaclematidis* based on phylogenetic analysis, and transferred *Immotthia* from Teichosporaceae to Dictyosporiaceae.

In our phylogenetic analysis, *Pseudocoleophoma* was divided into three clades (Fig. 1). Clade I comprised ten species (including *P.paraphysoidea*), but with lower support in the phylogenetic tree. Clade II comprises two isolates (ZHKUCC 22-0205 and ZHKUCC 22-0204) of *P.puerensis* and closely related to *Pseudoconiothyrium* (Fig. 1). Furthermore, Clade III is composed of *P.typhicola* and *P.guizhouensis* (Fig. 1). The confusing relationship of these three clades to *Immotthia*, *Verrucoccum*, *Pseudoconiothyrium*, and *Pseudocyclothyriella* demonstrates that *Pseudocoleophoma* is polyphyletic, which are consistent with previous studies (Dong et al. 2023; Zhang et al. 2023).

Morphologically, the species in clade I had morphological features typical of *Pseudocoleophoma*. Clade II includes only one species, *P.puerensis*, which has been reported to have a sexual morphology that is distinct from members of *Pseudocoleophoma* due to its brown spores (Lu et al. 2022). *Pseudocoleophomatyphicola* in clade III is different from members of *Pseudocoleophoma* in its septate conidia (Hyde et al. 2016), but *P.guizhouensis*, a sister clade, has been reported as a sexual morph, which are consistent with *Pseudocoleophoma*. Further research is needed to elucidate the relationship among *Pseudocoleophoma*, *Immotthia*, *Verrucoccum*, *Pseudoconiothyrium*, and *Pseudocyclothyriella*.

## Supplementary Material

XML Treatment for Gregarithecium bambusicola

XML Treatment for Pseudocoleophoma paraphysoidea

## ﻿Additional information

### Author ORCIDs

Yao Feng https://orcid.org/0000-0002-0888-8775

Zuo-Yi Liu https://orcid.org/0000-0001-5348-8458

Xiao-Fang Chen https://orcid.org/0009-0000-9962-7644

Mi-Lian Yang https://orcid.org/0000-0003-3511-3630

Zhi-Yuan Zhang https://orcid.org/0000-0003-2031-7518

Ya-Ya Chen https://orcid.org/0000-0002-8293-168X

### Data availability

All of the data that support the findings of this study are available in the main text.
